# Dynamic clamping induces rotation-to-beating transition of pinned filaments in gliding assays

**DOI:** 10.1098/rsif.2024.0859

**Published:** 2025-05-07

**Authors:** Amir Khosravanizadeh, Serge Dmitrieff

**Affiliations:** ^1^ Institut Jacques Monod, Université Paris Cité, Paris, France

**Keywords:** dynamics, cytoskeleton, actin, motor, mechanics, buckling

## Abstract

We used numerical simulations to investigate how properties of motor proteins control the dynamical behaviour of driven flexible filaments. A filament on top of a patch of anchored motor proteins is pinned at one end, a setup referred to as a spiral gliding assay. There exists a variety of motor proteins with different properties. We found that when these properties are changed, this system generally can show three different regimes: (i) fluctuation, where the filament undergoes random fluctuations because the motors are unable to bend it, (ii) rotation, in which the filament bends and then moves continuously in one direction, and (iii) beating, where the filament rotation direction changes over time. We found that the transition between fluctuation and rotation occurs when motors exert a force sufficient to buckle the filament. The threshold force coincides with the second buckling mode of a filament undergoing a continuously distributed load. Moreover, we showed that when motors near the pinning point work close to their stall force, they cause dynamic clamping, leading to the beating regime. Rather than being imposed by experimental conditions, this clamping is transient and results from the coupling between filament mechanics and the collective behaviour of motors.

## 1. Introduction

In living cells, actin filaments and microtubules are two major cytoskeletal filaments. Actin is a double helical filament with a radius of a few nanometres and lengths that can reach hundreds of nanometres [1] *in vivo* and micrometres *in vitro* [2], classifying it as a thin filament. Actin is a semiflexible polymer with a persistence length of up to 17 μm [3]. Microtubules are hollow, rigid rods with a persistence length of a few millimetres [3,4]. The radius of microtubules is approximately 25 nm, while their lengths can extend to several micrometres [5]. Despite their persistence length, both types of filaments can bend on (sub-)micrometric length scales, because of external loads, including the activity of motor proteins (here referred to as motors) [6,7]. Motors hydrolyse adenosine triphosphate (ATP) to move directionally along the associated filaments; motors called myosins walk onto F-actin filaments, while kinesins and dyneins are associated with microtubules [8]. There exist dimeric motors that can exert forces on two filaments and rearrange them or form a network [9,10]. One remarkable example is that of cilia, made of 9 + 2 microtubule doublets linked by minus end-directed dynein motor proteins. Owing to the activity of the dyneins, the doublets bend and generate a continued self-organized beating motion [11]. *In vitro* experiments show that actin bundles with dimeric myosins also exhibit beating behaviour, suggesting a universal property of bundled filaments with motors [12]. However, single filaments driven by monomeric motors can also exhibit beating in certain conditions [13].

A common assay to assess motor properties is the gliding assay, in which motors grafted to a coverslip can apply tangential forces to microfilaments in the presence of ATP and slide them across the surface [14–18]. However, defects on the surface or inactive motors can act as a pinning point and fix one end of the filament [2,19,20]. In this situation, filaments can exhibit a rich variety of dynamical behaviour, such as spiralling, swirling and beating [19,21–24]. Early gliding assays were able to measure the buckled shape of the filaments to estimate the average force exerted by motors [19], and continuum elastic models have been applied to determine the shape of the filament as a function of motor forces [25,26].

There are various motors with different characteristic properties such as length, step size, velocity, stall force, binding rate and unbinding rate [27,28]. While some studies have examined the influence of motor density and filament length [13], the impact of motor properties remains unexplored. For example, the dynamics of the filament can be significantly influenced by how the attachment–detachment rate depends on external forces and time.

In this study, we have used numerical simulations to explore the impact of motor properties on the dynamical behaviour of a spiral assay. In §2, we present the details of the numerical model. The results of the simulations are summarized in §3, where we have found three distinct regimes in the phase diagram of the system: (i) fluctuating regime, where the filament undergoes random fluctuations because motors are not attached to the filament long enough to bend it, (ii) rotation regime, characterized by the bending of the filament as it moves continuously in either a clockwise or anticlockwise direction, and (iii) beating regime, in which the filament is moving continuously, but the rotation direction frequently reverses.

We propose a simple explanation for the phase transitions between these three different regimes, based on motor properties. The fluctuation–rotation transition happens when motors overcome the filament’s buckling force, albeit under a continuous load. Moreover, the motors close to the pinning point are responsible for the transition between the rotation and beating regimes. In this region, if motors operate close to, or above, their stall force, they stall and can act as a second pin, fixing the direction of the filament and leading to its beating.

## 2. Model

The simulations presented in this work are based on a highly coarse-grained description of the cytoskeletal filaments. For this purpose, we use Cytosim [29,30], an open-source package based on overdamped Langevin dynamics.

**Figure 1.**
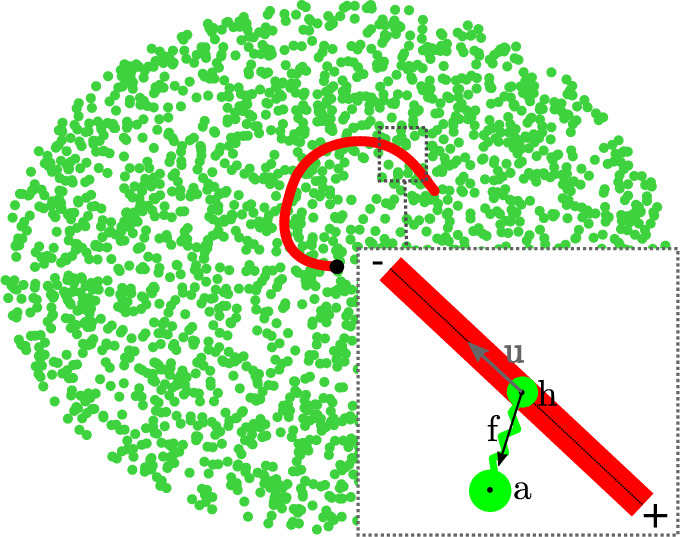
A thin filament (red) is pinned at one end (black dot) positionally but not directionally. Motor proteins in green are anchored to the surface and attempt to walk towards the free (plus) end of the filament. Inset: motors move towards the filament plus (+) end. When they extend, they exert a force $f=-k_{\text{m}}(h-a)$ on the filament, with $k_{\text{m}}$ the motor stiffness.


We consider a non-extensible elastic filament represented by N segments and N+1 point vertices. Each segment has a fixed length L/N, where L is the total length of the filament. The bending energy per unit length of the filament is $1/2\kappa C^{2}$, where κ and C are the bending rigidity and curvature of the filament, respectively. This energy can be written in a discrete form, and forces on the vertices can be derived from it. Movement of vertices then follows the overdamped Langevin equation [29]. Forces that are assumed to derive from locally quadratic potentials can be linearized locally. This allows to solve vertex displacement through a semi-implicit algorithm by diagonalizing a matrix. The semi-implicit algorithm leads to higher stability of stiff problems, allowing the use of larger timescales than explicit algorithms. The filament is polar, with its minus end fixed at a central position, but the filament still can rotate around the fixed point (see figure 1). The filament is placed on top of a substrate of anchored motors, which are randomly distributed within a circular patch of radius L. The motors can bind along the filament (not only on vertices) within a binding range $r_{\text{b}}$ at a constant binding rate $\omega_{\text{on}}$. Motors are modelled as elastic springs of stiffness $k_{\text{m}}$; extension of these springs results in a motor force f following a Hookean law: $f=-k_{\text{m}}(h-a)$, with a the position of the motor anchor and h the position of the motor head bound to the filament. Bound motors can detach from the filament with an off-rate $\omega_{\text{off}}$, that depends on both time and the resistive load:

(2.1) $$\omega_{\text{off}}=\omega_{\text{d}}\exp(\|f\|/f_{\text{d}}),$$

where $\omega_{\text{d}}$ is a constant detachment rate and $f_{\text{d}}$ is the typical unbinding force. Motor detachment follows a Gillespie algorithm [31], modified for variable reaction rates: when a motor is attached, a (normalized) timer is drawn from an exponential distribution to compute the next detachment time. Then, at each timestep, the timer is decreased by $w_{\text{off}}\text{d}t;$ detachment happens when the timer is negative [32]. On the other hand, motor attachment follows an Euler algorithm: at each timestep, motor heads bind to a filament (within range $r_{\text{b}}$) with a probability $\omega_{\text{on}}\text{d}t$.

An attached motor tends to move along the filament towards the plus end. The motors implemented in this study are continuous: they do not have a finite step size but rather move at a velocity $v_{\text{m}}$. This velocity decreases with the projected load $f^{T}$ against the movement, following the relationship


(2.2) $$v_{\text{m}}=v_{0}\left(1-\frac{f^{T}}{f_{\text{s}}}\right)$$

in which $v_{0}$ is the motor unloaded speed, $f_{\text{s}}$ is its stall force and $f^{T}$ is the projection of the motor force in the local tangential direction u of the filament towards the minus end: $f^{T}=f\cdot u$. Thus, it is also possible for motors to move towards the minus end if they are being pulled with a tangential force $f^{T}$ larger than the stall force, $f_{\text{s}}$. At each time step dt, the speed $v_{\text{m}}$ is computed through equation (2.2), and the motor is moved along the filament by a displacement $\delta s=v_{\text{m}}\text{d}t$.

In this study, we chose to model actin filaments and myosin motors, with parameters chosen in the range of experimental values. The simulation parameters are summarized in table 1 unless mentioned otherwise. We initially run the simulations for 5000 time steps to reach a steady state. Following that, we continue for $3\times 10^{5}$ time steps, equivalent to 15 s, while saving frames every 0.15 s.

**Table 1.** Simulation parameters.

symbol	parameter	value
$k_{\text{B}}T$	thermal energy	4.2pNnm
Δt	time step	$5\times 10^{-5}\;\text{s}$
V	box volume	$2\;\times \;2\;\times \;0.1\;{\text{μm}}^{3}$
filament		
*L*	filament length	1μm
κ	bending rigidity	$0.075\;\text{pN}\;\mu {\text{m}}^{2}$ [3]
d*s*	segmentation length	10nm
motors		
$N_{MP}$	number of motors	$1.5\times 10^{4}$
$\omega_{\text{on}}$	binding rate	$10\;{\text{s}}^{-1}$ [33]
$r_{\text{b}}$	binding range	5nm
$f_{\text{s}}$	stall force	5pN [34]
$v_{0}$	unloaded speed	$2\;\mu \text{m}\;{\text{s}}^{-1}$ [35]
$k_{\text{m}}$	spring stiffness	$500\;\text{p}\text{N}\;\mu {\text{m}}^{-1}$

## 3. Results

### 3.1. Actin filaments glide as spirals

**Figure 2.**
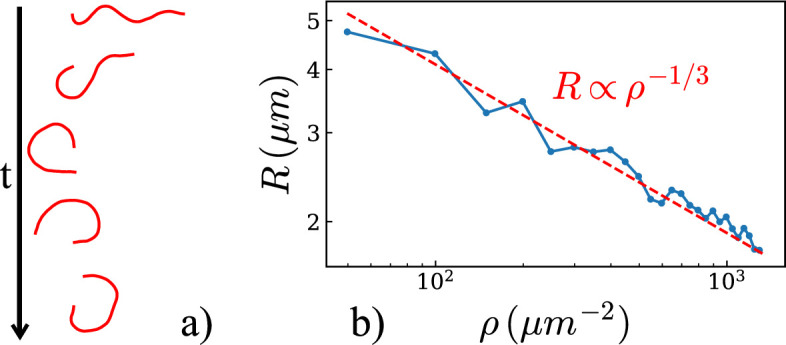
(a) The time-lapse shows a filament of length 8μm forming a spiral over time. (b) Variation of the spiral radius as a function of motor density. The radius decreases following the relation $R\propto \rho^{-1/3}$, as predicted in [19].

We first investigated the dynamics of an actin filament with a single pinning point on a bed of motors with L=8μm, $\omega_{\text{d}}=20\;{\text{s}}^{-1}$ and $f_{\text{d}}=4\;\text{p}\text{N}$. We modelled long filaments, similar to those often used in *in vitro* experimental assays [2]. We observed that at sufficient motor densities, the filament adopted a spiral shape (figure 2a) and displayed a rotational motion (electronic supplementary material, video S1, with $\rho =500\;{\text{μm}}^{-2}$). By calculating the mean radius of curvature of the filament, we found that it decreased with motor density (figure 2b), as described in previous work [19]. For some values of $\omega_{\text{d}}$ and $f_{\text{d}}$, we observed that the direction of rotation could change over time (electronic supplementary material, video S2, for $\omega_{\text{d}}=8\;{\text{s}}^{-1}$, $f_{\text{d}}=4\;\text{p}\text{N}$ and $\rho =500\;{\text{μm}}^{-2}$). This is similar to the beating of filaments observed experimentally [2,19]. At high motor densities, the radius of the spiral reduces, and it adopts a complete circular shape (electronic supplementary material, video S3), but some values of $\omega_{\text{d}}$ and $f_{\text{d}}$ still lead to a change in the direction of rotation (electronic supplementary material, video S4).

For all motor densities, this change of direction seemed to originate from the pinned end. In experiments, it was claimed that a defect close to the pinning point, or an extended pinning area, could lead to beating rather than pure rotation [2,19]. In our system, there are neither secondary anchors nor inactive motors, and this beating was not expected.

### 3.2. Systematic simulations of short filaments

**Figure 3.**
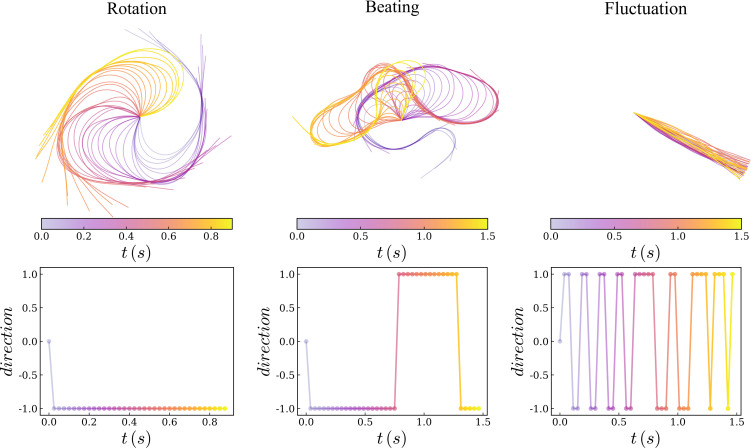
Illustration of the three regimes. The top panels illustrate the typical movement of the filament over time, while the bottom graphs display rotation direction as a function of time. In this representation, −1 corresponds to clockwise movement and +1 represents anticlockwise rotation.

**Figure 4.**
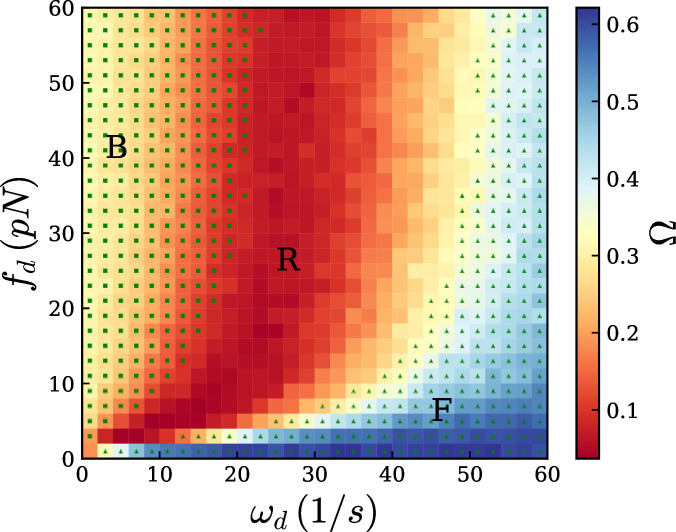
Phase diagram where colour indicates rotation direction change frequency (Ω, denoting the number of changes in the rotation direction of the filament) as a function of detachment rate ($\omega_{d}$) and detachment force ($f_{\text{d}}$). There are three distinct regimes: fluctuation (F), rotation (R) and beating (B). Squares and triangles are correspond to the beating and fluctuation regimes, respectively, predicted from our hypotheses.

Very long filaments such as those modelled above are commonly used in gliding assays and single-filament *in vitro* experiments because their large size makes them easier to image. However, disentangling the mechanics behind the rotation direction changes in such long filaments is challenging; moreover, actin filaments *in vivo* are typically shorter [1]. Therefore, we decided to simulate shorter filaments of 1μm in length. This size is achievable *in vitro* [36,37], with sufficient resolution [37], and can be simulated efficiently over long timescales. Because we saw the change of direction originate from the pinned end, we hypothesized that rotation direction change would also be observed with shorter filaments. We used an experimentally relevant set of parameters (table 1), and we varied the detachment rate ($\omega_{\text{d}}$) and the detachment force ($f_{\text{d}}$), to measure the rotation direction change frequency (Ω). Using the direction of the filament $u_{i}$ (at an arbitrary point i) over time, it is straightforward to calculate the direction d of the rotation by the direction of the vector product $d=\hat{z}.\left[u_{i}(t)\times u_{i}(t+dt)\right]$, with $\hat{z}$ the director vector normal to the plane. The direction is thus −1 for clockwise movement and +1 for anticlockwise movement. We chose i=30, i.e. a point 300 nm away from the pinned end, to compute the direction at different times, figure 3, bottom. For instance, the rotation direction changes twice in the beating regime and 16 times in the fluctuation regime in figure 3. The rotation direction change frequency (Ω) is defined as the number of changes in the direction of the rotation divided by the total number of frames (here 100 frames). If the filament constantly rotates in one direction Ω is close to zero, and if the direction changes in each frame, it reaches Ω=1. The results of 22 500 simulations for different values of $\omega_{\text{d}}$ and $f_{\text{d}}$ are displayed in figure 4, where colours represent Ω.

**Figure 5.**
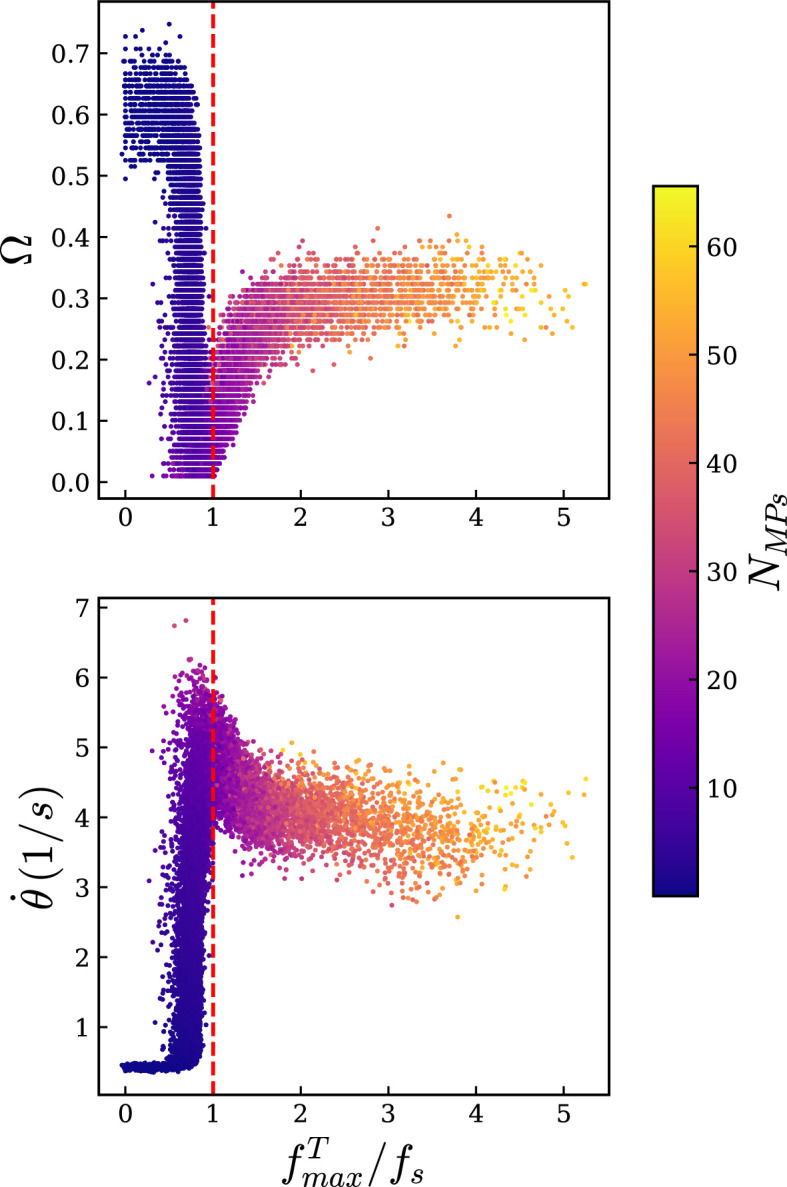
The rotation direction change frequency (top) and the speed of rotation (bottom) of the filament as a function of the maximum tangential force. A phase transition between the rotation and beating regimes happens around $f_{\text{max}}^{T}=f_{\text{s}}$. The colour bar denotes the average number of bound motors.

To reduce the stochasticity of the individual results in the total phase diagram, we averaged individual simulation results with neighbouring simulations (close in the $f_{\text{d}},\omega_{\text{d}}$ parameter space). The original data of individual simulations is presented in electronic supplementary material, figure S1. Because the simulated filaments are not chiral, there is no favourite direction of motion; see electronic supplementary material, figure S2. The phase diagram shows three different regimes (figure 3 and electronic supplementary material, video S5). For a large detachment rate $\omega_{\text{d}}\gg 1$, or a very small detachment force $f_{\text{d}}≃0$, motors cannot stay attached to the filament long enough ($\omega_{\text{off}}\gg 1$) to apply a sufficient force to the filament to bend it. This regime is named ‘fluctuation’ (F) where the filament merely jiggles while remaining mostly straight. Conversely, for small $\omega_{\text{d}}$ and large $f_{\text{d}}$, the system tends to continuously ‘beat’ (B), wherein it rotates in one direction and then changes to another. In between, the system exhibits a continuous ‘rotation’ (R), with few or no changes of direction. We also found that the rotation speed changes slowly between the rotation and fluctuation regimes, while it is much lower in the fluctuation regime (figure 5 and electronic supplementary material, figure S3).

### 3.3. Fluctuation to rotation

We hypothesized that the transition from the fluctuation regime to rotation happens when motor proteins can exert enough force to overcome the buckling force of the filament. Indeed, we could observe a continuous increase in the number of bound motors between the fluctuation and the rotation regime, electronic supplementary material, figure S4, corresponding to an increase in the filament bending energy, electronic supplementary material, figure S5. Here, the force is applied by motors all over the filament length, rather than on the ends only, and the buckling threshold should differ from the canonical Euler buckling threshold $f_{\text{B}}=\pi^{2}\kappa /L^{2}$. To model the motor distribution, we assumed a force density w. Up to buckling, the filament is straight, and we can discard feedback between filament mechanics and motor force, and thus we can assume w to be constant. Similar to Euler buckling, we can solve the Euler–Lagrange equations and find the filament shape; see electronic supplementary material. The buckling threshold is found by computing the first modes that satisfy the boundary conditions. The main difference with Euler buckling is that the force on the free end is 0, so we can compute the threshold force at the pinned end. This is very similar to the buckling of a column under gravitational load [38], albeit with slightly different boundary conditions. We find that the critical force at the pinned end is $f_{j}^{*}={\bar{w}}_{j}\frac{\kappa }{L^{2}}$, with ${\bar{w}}_{j}$ the critical force density of mode j: $\bar{w}\approx \left[25.64,95.95,210.68,369.828,\ldots \right]$; note that this force is at least ∼2.5 times larger than $f_{B}$. We should expect the F→R transition to occur when the sum of motor forces along the filament is larger than $f_{1}^{*}$.

To obtain this result, we assumed the filament shape to be controlled by its rigidity. However, because motors act as Hookean springs, they could also restrict the displacement normal to the filament. If motor distribution were continuous, this would result in adding an elastic potential normal to the filament axis. There is no straightforward solution to the buckling problem with a continuous force along the filament axis and a quadratic potential in the normal axis, but it is clear that the normal potential could strongly increase the buckling force, with or without selecting higher modes [39]. On the other hand, in the limit of high motor stiffness, discrete motors could act as additional constraints on the solutions (similar to boundary conditions), rather than as a potential. In that case, they could select higher modes of deformation for filaments [40], and thus increase the critical load density. We measured the total force exerted by motors on the filament in simulations, and we found that the buckling threshold happens to coincide with the total motor force being larger than $f_{2}^{*}$, the second deformation mode of a filament under a constant load density: in figure 4, triangles represent the area where the total force is smaller than $f_{2}^{*}$.

### 3.4. Rotation to beating

The second transition is between the rotation and beating regimes, both of which have been observed in experiments. Theoretical work showed that pinning the filament via a second anchor leads to beating [13,19,25]. In contrast, in our simulations, we have a single pinning point rather than a clamped end, and this beating was a surprise. In experiments exhibiting beating, it was claimed that this behaviour occurs when there is a second defect close to the pinning point or the pinning point extends to a line [2,19]. In our simulations, we did not include secondary anchors or inactive motors; yet, we could observe beating. As for long filaments, the change of rotation direction originated from the pinned end of the filaments, as visible from plotting the local curvature along the filament and over time (electronic supplementary material, figure S6). Therefore, we wondered if active motors could transiently act as a clamp, for example, if they were driven beyond their stall force. We therefore decided to compute the mean number of motors, and their tangential force, as a function of the arclength. We averaged this value over time, and over a hundred simulations to reduce the stochasticity of the results.

**Figure 6.**
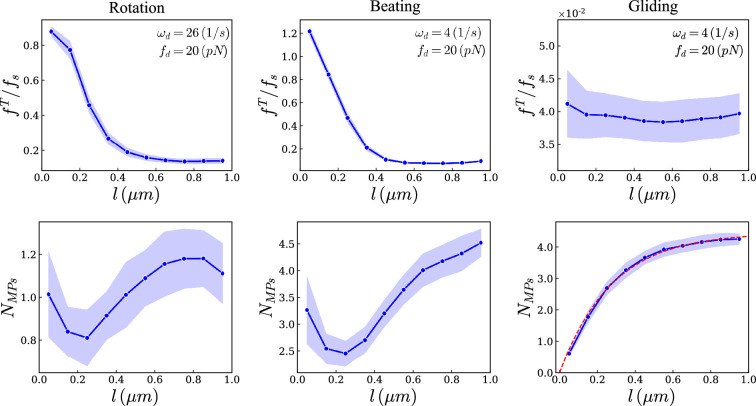
At the top, the magnitude of the tangential force is shown as a function of contour length along the filament for the rotation and beating regimes, as well as a gliding assay without any pinning point as a control. In the rotation and gliding regimes, the motors never reach forces higher than the stall force. In contrast, in the beating case, motors near the pinning point can exert forces exceeding their stall force, effectively acting as a second pin. At the bottom, the number of motors is shown as a function of contour length along the filament. The filament is divided into ten equal segments, and both the force and the number of motors are averaged over time for each segment. To minimize stochastic effects, the plots are averaged over 100 simulations.

First, as a control, we computed this tangential force in an unpinned gliding assay, in which the filament is free to slide on a surface of anchored motors. We were able to formulate a master equation to predict the motor density in such a gliding assay; see electronic supplementary material. Motors were assumed to bind all along the filament, and move along the filament. Because the filament is not pinned, we could assume the motor force to be independent of position; therefore, the motor velocity and unbinding rate should be independent of position. We therefore predicted an exponential motor distribution as a function of arclength (figure 6, right). In simulations, we do find the force per motor to be near-independent of position; accordingly, we find a near-exponential distribution for the motor density. We also find that the force exerted by motors is only a fraction of the motor stall force: if the filament drag is low enough, the motor operates near its maximum speed at low forces. Indeed, changing systematically motor on/off rates showed that filament velocity quickly saturated to $v_{0}$ when enough motors were bound; electronic supplementary material, figure S7.

In contrast, for a pinned filament, we find a non-monotonic motor density as a function of arclength (figure 6, left and middle). While we expect motor binding to be independent of position, forces applied by motors depend on their positions along the filament (figure 6, lower panels). Therefore, the motor velocity and unbinding rate should depend on position, yielding a coupling between filament mechanics and motor dynamics. In the rotating case (figure 6, left), the force exerted by motors is higher than for a gliding assay because motors have to exert more force to bend the filament than to merely cause a translational motion. The force is higher close to the pinned end of the filament but remains below the stall force.

In contrast, in the beating case, the tangential force near the pinned end is higher than the stall force. As an isolated motor cannot exert a force beyond its stall force, this is due to the other motors on the filament deforming the filament and thus stretching the motors near the pinned end at forces higher than the stall force. As a consequence, motors close to the pinned (minus) end are being dragged towards the pinned end rather than walking towards the free (plus) end; this results in an accumulation of motors at the pinned end, that are moving in reverse. We believe that this accumulation of motors beyond their stall force is causing the beating. Individual simulations confirm this accumulation in the beating case (electronic supplementary material, figure S8) and its absence in the rotation case (electronic supplementary material, figure S9).

Accordingly, a transition from rotation to beating should occur when the tangential force along the filament surpasses the stall force. In simulations, we computed the maximal tangential force along the filament, averaged over time $f_{\text{max}}^{T}$; we found that simulations with $f_{\text{max}}^{T}>f_{s}$ mostly belonged to the beating phase rather than the rotation space (figure 4). Using these simulation results and plotting the rotation direction change frequency of the system as a function of $f_{\text{max}}^{T}$ indeed revealed a phase transition occurring around $f_{\text{max}}^{T}=f_{\text{s}}$, (figure 5).

**Figure 7.**
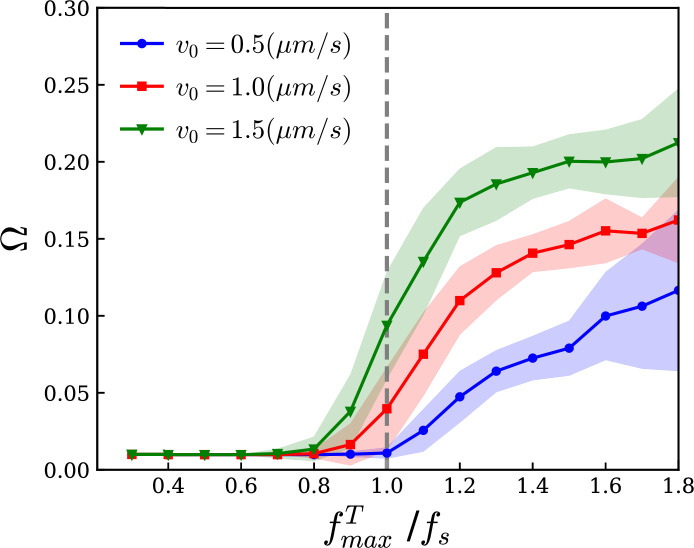
The rotation direction change frequency as a function of the maximum tangential force for three different unloaded speeds, $v_{0}=0.5,1.0,1.5\;({\text{μm s}}^{-1})$. A phase transition between the rotation and beating regimes occurs around $f_{\text{max}}^{T}=f_{\text{s}}$.

To confirm that the rotation to beating transition was caused by motors near the pinned end acting as additional anchors, as the filament is being moved by the other motors on the filaments, we decided to explore the behaviour of the system as a function of two other motor parameters. We systematically varied the motor stall force $f_{\text{s}}$, for several values of the unloaded speed $v_{0}$. We found that Ω increases drastically above a threshold of $f_{\text{max}}^{T}/f_{\text{s}}$ close to 1, indicating the rotation to beating transition (figure 7). The precise threshold of $f_{\text{max}}^{T}/f_{\text{s}}$ is shifted by approx. 20% while $v_{0}$ is changed by approx. 300%. Therefore, while other parameters play a minor role, the rotation to beating transition is mostly driven by $f_{\text{max}}^{T}$ being larger than $f_{\text{s}}$. We decided to coin this behaviour ‘dynamic clamping’, as motors near the pinned end act transiently as a clamp, before stochastically detaching.

## 4. Discussion

We found three different regimes for the spiral gliding assay: (i) the fluctuation (F) regime, where motors do not exert enough force to bend the filament, whereby the filament jiggles around its position, (ii) the rotation regime (R), characterized by the filament bending and continuously rotating in one direction, and (iii) the beating regime (B), wherein the filament rotates but the direction of rotation frequently reverses, alternating between clockwise and anticlockwise.

We found that the F→R transition is a consequence of the motors collectively exerting a force higher than the buckling force of the filament with a transition for a total force larger than $f_{2}^{*}∼95.95\kappa /L^{2}$. This is approximately 10times larger than the Euler buckling threshold $f_{\text{B}}=\pi^{2}\kappa /L^{2}$. The buckling threshold $f_{2}^{*}$ coincides with the second buckling mode of a pinned filament driven by a constant motor density that we were able to compute analytically. However, this high buckling force could also stem from the confinement by motors of the filament along its normal direction.

Surprisingly, we also find a R→B transition, from rotation to beating, although the filaments have a single pinning point. We proposed that the R→B transition results from the motors near the pinning point, which effectively act as a second pin that transiently fixes the local filament direction, leading to its beating as predicted for a clamped filament, a process we coined ‘dynamic clamping’. Our simulations confirmed this hypothesis. Motors at the pinned end operate close to, or above, their stall force, moving slowly or even backwards on the filament, thus accumulating at the pinned end and clamping the filament. This was confirmed by changing the motor stall force, showing that the R→B transition is mostly controlled by the $f_{\text{max}}^{T}/f_{\text{s}}$ ratio; above a threshold value close to 1, the rotation direction change frequency (Ω) strongly increased in a stepwise manner.

In a gliding assay without any pinning point, motors never exceed their stall force, while in a spiral system, motors near the pinning point can reach forces even higher than their stall force. This highlights an unexpected coupling between filament mechanics and collective motor action. Accordingly, both transitions are strongly controlled by the motor properties, both motor unbinding rate $w_{d}$, unbinding force $f_{\text{d}}$, stall force $f_{\text{s}}$ and unloaded velocity $v_{0}$, in a non-trivial way.

Dynamic clamping, and hence the R→B transition, were not identified in previous theoretical works. In some works, a clamped end (without orientational degree of freedom) was used instead of a pinned end [13]. In other works, a single pinned end was used, but no beating was observed as neither the force dependence of motor velocity nor the discrete nature of motors were included [25]. Here, we found that the coupling between filament mechanics and motor properties plays a crucial role. We thus identified a novel behaviour for a simple two-component system. This could be tested experimentally with existing, well-established experimental setups [36]. While we mostly focused on actin filaments, this behaviour is not actin-specific and is also expected to take place for microtubules, albeit for higher motor densities, because of the higher filament rigidity.

While gliding assays of single filaments are an over-simplification of biological instances of the cytoskeleton, the strong control by motor properties reveals a crucial property of cytoskeletal networks. While the existence of filament rotation and beating phases are universal consequences of filament–motor assemblies, the phase in which the system lives depends strongly on motor properties, and thus on which precise motor proteins exist locally. Therefore, local signalling pathways (such as Rho, Rac and Cdc42 for actin [41]) allow the cell to explore various areas of the phase diagram by recruiting a few specific motors and cytoskeleton-associated molecules.

## Data Availability

A configuration file with the parameters used in this article is available as electronic supplementary material [42].
